# Maize variety traits for different needs: using the means-end chains method to identify preferences and perceived benefits among smallholder farmers in Kenya

**DOI:** 10.1007/s12571-025-01637-2

**Published:** 2026-02-12

**Authors:** Mariana Garcia-Medina, Fleur Kilwinger, Conny Almekinders, Jason Donovan

**Affiliations:** 1 Knowledge, Technology and Innovation Group, Wageningen University & Research, Hollandseweg 1, Wageningen, 6706 KN Netherlands; 2 Copernicus Institute of Sustainable Development, Utrecht University, Vening Meinesz building, Princetonlaan 8a, Utrecht, 3584 CB The Netherlands; 3 Sustainable Agri-food Systems Program, International Maize & Wheat Improvement Center (CIMMYT) Carretera México-Veracruz, Km. 45 El Batán, Texcoco, 56237 México; 4 International Development Research Centre (IDRC), Torre Mapfre, Juncal 1385, Montevideo, 11000 Uruguay

**Keywords:** Maize attributes, Crop breeding, Gender, Social differentiation, Varietal turnover

## Abstract

Maize breeding programmes have developed a new generation of hybrid varieties to improve smallholders’ productivity and enhance climate resilience. However, varietal turnover in Kenya remains low, suggesting that new hybrid maize varieties may not fully address smallholders’ needs or that knowledge about them remains limited. In this exploratory study, we applied a method referred to as means-end chains to understand the attributes smallholders consider when differentiating maize seed products, and the importance and value of these attributes. We interviewed 82 smallholders in two counties in Kenya and analysed the data by county and gender. Smallholders used a range of attributes to differentiate maize seed products, indicating familiarity with most maize varieties included in the study. However, the attributes that farmers used to distinguish between maize seed products were not always those of highest value when choosing seeds for planting. Preferences for attributes differed between counties and were shaped by climate and the importance of maize in livelihoods. Women and men used and preferred similar attributes, yet their choices were informed by different underlying motivations and values. Overall, participants highly valued ‘higher yield’, ‘harvest assurance’ and ‘earliness’, reflecting diverse household uses of maize to support food security, income generation and well-being. The findings suggest that farmers use a portfolio of maize varieties to meet different household needs. These results have implications for efforts to promote varietal turnover and complement previous studies by offering guidance for demand-led breeding programmes and other seed systems actors working to strengthen food security for smallholder farmers.

## Introduction

Maize is one of the three most important food crops in the world and ranks second after wheat in cultivated area (FAO, 2025). The grain is important for food security by providing multiple dietary components that can help address the triple burden of undernutrition, micronutrient malnutrition, and overnutrition (Poole et al., 2021). Over the past decades, governments and donors made considerable investments in breeding hybrid maize varieties for regions such as Sub-Saharan Africa, where maize is crucial for both food security and economic development (Mango & Hebinck, 2004; Rutsaert & Donovan, 2020b; Walker & Alwang, 2015). In recent years, a new generation of hybrid maize varieties has been developed by public and private, national and international breeding programmes to address current and emerging cultivation challenges. For instance, over 80 stress-tolerant hybrid maize varieties were released in Kenya during the past 15 years (De Groote & Omondi, 2021; Rutsaert & Donovan, 2020b).

With the release of this new generation of hybrid maize varieties, discussions have shifted from the adoption of improved varieties to variety turnover (De Groote et al., 2025; Spielman & Smale, 2017). Varietal turnover refers to the replacement of an old variety with a more recently developed one (Brennan and Byerlee, 1991; De Groote et al., 2025; Spielman & Smale, 2017). Breeding programmes that developed new hybrid maize varieties have focused on agronomic attributes such as higher yields and yield stability under stress conditions, including drought, heat, and low nitrogen availability, as well as resistance to maize streak virus, maize lethal necrosis, and striga (Cairns & Prasanna, 2018). Breeding efforts have also included end-use attributes such as grain colour, grain texture, and (through biofortification) increased the levels of provitamin A, lysine, tryptophan, and zinc (Cairns et al., 2021; Ekpa et al., 2018). Nevertheless, the observed increase in maize production in sub-Saharan Africa (SSA) is associated with an expansion in maize area rather than by yield gains (Abate et al., 2017; Cairns et al., 2021). Abate et al. (2017) and Ray et al.(2012) explain the stalled maize productivity in SSA partly by the slow adoption of the new generation of hybrid maize varieties by smallholder farmers. Atlin et al. (2017) documented a low maize varietal turnover rate in the region, with farmers growing hybrid maize varieties that are over 20 years old (Abate et al., 2017). Farmers in Kenya are continuing to purchase hybrid maize varieties older than 10 years (Makinde & Muhhuku, 2017; Odame & Muange, 2011; Rutsaert & Donovan, 2020b).

Researchers present a range of factors along the seed value chain that contribute to a slow varietal turnover. Voss et al. (2021) indicate that breeding programmes may not yet sufficiently address smallholders’ production and consumption preferences and needs. Others point to suboptimal agronomic practices, such as low fertiliser use, and limited access to irrigation, input supply, and extension services that limit farmers’ ability to benefit from the genetic potential of new hybrid varieties. (Atlin et al., 2017; Challinor et al., 2016; Erenstein & Kassie, 2018; Rutsaert et al., 2021, 2024; Rutsaert & Donovan, 2020b; Spielman & Smale, 2017). Research has also shown that (1) maize seed companies maintain old hybrid maize varieties in Kenya’s market, while (2) smallholders are often considered risk-averse or insufficiently informed about available, new hybrid maize varieties (De Groote & Omondi, 2021; Rutsaert et al., 2021; Rutsaert & Donovan, 2020a). Recent studies pointed out that there is still limited knowledge on the variation in preferences among different smallholder groups and contexts (Voss et al., 2021; Almekinders et al., 2021a), which can result in underestimating the importance of local maize varieties (Almekinders et al., 2021b; Mango & Hebinck, 2004). In this regard, the positive attributes of local maize varieties in terms of taste, storage traits, price, and accessibility have received little attention in breeding programmes (Almekinders et al., 2021b; Voss et al., 2021). It therefore remains a main and relevant line of research to better understand the preferences of smallholder farmers for attributes of maize varieties and seeds.

Over the past two decades, many researchers examined smallholders’ preferences for maize seeds and variety traits using a range of research methods. Studies have mostly used surveys (Boakyewaa Adu et al., 2021; Hintzea et al., 2003; Lunduka et al., 2012; Nyaligwa et al., 2017; Smale et al., 2001), field trials for on-farm variety evaluations and participatory varietal selection (Bellon et al., 2003; De Groote et al., 2013; Mulatu & Zelleke, 2002; Tegbaru et al., 2020), group discussions (Dao et al., 2015; Nyaligwa et al., 2017), and choice experiments (Asrat et al., 2010; Kassie et al., 2017; Marenya et al., 2021, 2022; Mastenbroek et al., 2021; Sánchez-Toledano et al., 2017). We argue that these approaches have captured specific aspects of smallholders’ seed and variety preferences but have overlooked others. For example, on-farm trials allow farmers to make a thorough evaluation of varietal performance in the field but ignore attributes such as packaging and price. Ranking exercises provide insights into farmers’ priorities, but often for a limited, predefined set of attributes. Choice experiments frequently use hypothetical seed options rather than those that are available on the market. Acknowledging that each method has its own rationale and associated interpretation of the findings, and that no single method can capture all aspects of smallholders’ seed choices (Almekinders et al., 2019), we used in this study a means-end chain (MEC) analysis as an exploratory and complementary method to contribute to the understanding of smallholders’ variety and seed choices. By using means-end chains analysis, we aim to gain an in-depth understanding of the attributes that different smallholder groups use to evaluate and distinguish between maize seed products, capturing the relative importance of specific attributes and elucidating the underlying motivations and values shaping their preferences. In this study, and in line with the approach, “seed product” refers to the 2 kg packages of seeds of hybrid maize varieties, or to the plastic bags with approximately 2 kg of seed of open pollinated variety (OPV).

## Methods

### Study areas

**Fig. 1:**
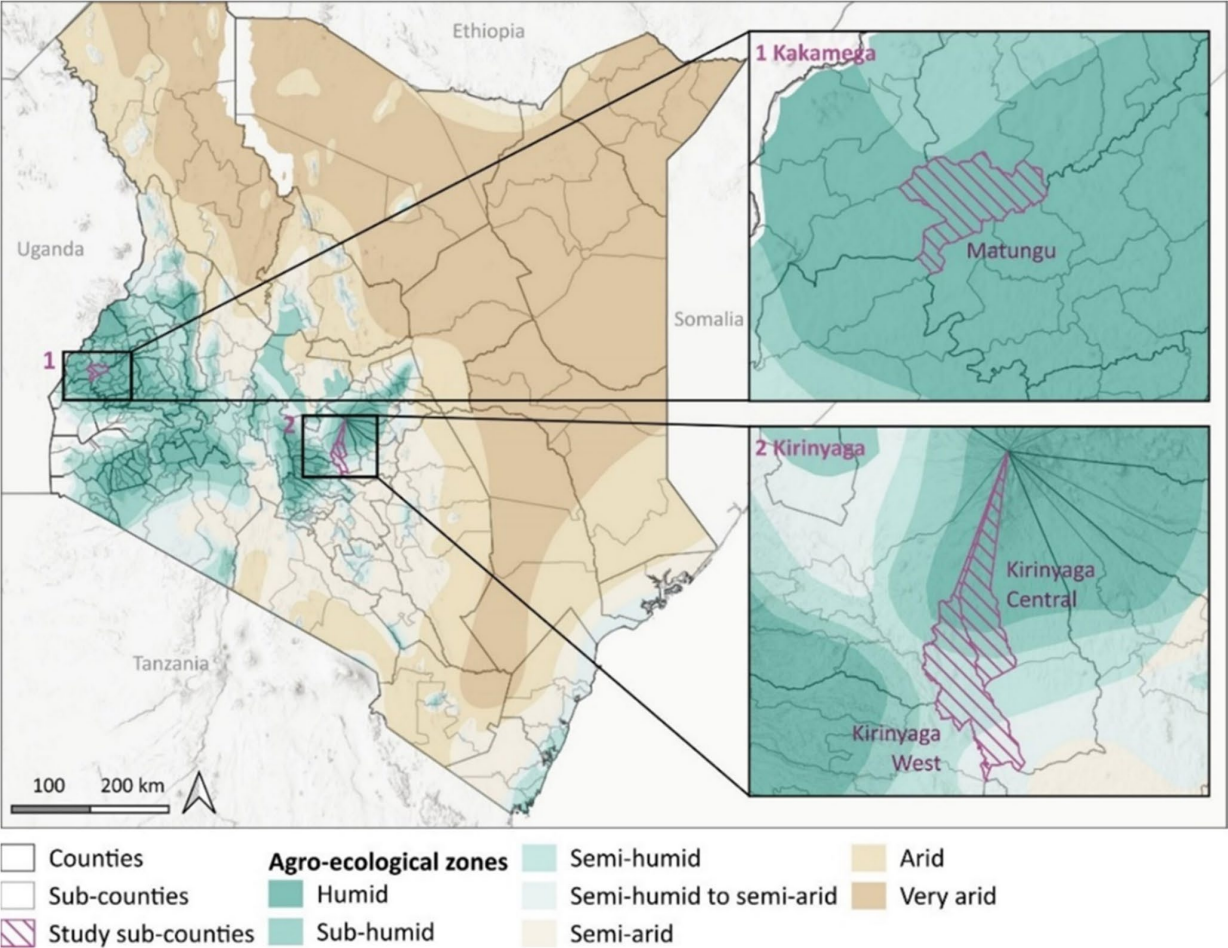
Map of study areas in Kenya

The study was conducted in two counties in Kenya: Kakamega and Kirinyaga, in the western and central parts of the country, respectively (Fig. 1). The counties were purposively selected based on their differences in climatic characteristics such as rainfall patterns influencing maize production (Table 1), proximity to the capital, and the role of maize in livelihoods and maize markets. Furthermore, those areas are important target areas for maize researchers and the formal maize sector in Kenya.

**Table 1:** Main characteristics of the study areas

County	Kakamega	Kirinyaga
Subcounty	Matungu	Kirinyaga West, Kirinyaga Central
Region	Southwest region	Western and Central regions
Ecological zone	Lower medium highland with a tropical rainforest climate	Lower highlands moist transitional altitude
Annual average temperature (°C)	18–29	10–25
Annual average precipitation (mm)	1281–2214	700–1400
Altitude (m)	1250–1380	1200–1600
Long rainy season	April to September	March to May
Short rainy season	September/October to March	October to December
Average small scale farm size (ha)	1.5	1.0
Average large scale farm size (ha)	10.0	5.2

*Data from: (County Government of Kakamega, 2018, 2023; County Government of Kirinyaga, 2018; Google, 2024; The Kenya Ministry of Agriculture Livestock and Fisheries, 2018a, 2018b)

In Kakamega County, the study took place in the southwest region, roughly 390 km from Nairobi. In 2019, 35% of the people in Kakamega lived below the poverty line (Kenya National Bureau of Statistics, 2022). Seventy-two per cent of the population lived in rural areas (The Kenya Ministry of Agriculture Livestock and Fisheries, 2018a) and in 2016, 21% of households were food insecure (World Food Program, 2016). The economy of Kakamega County is based on subsistence agriculture (The Kenya Ministry of Agriculture Livestock and Fisheries, 2018a).Maize is usually planted twice per year, mostly under rainfed conditions and inter-cropped with beans and other local vegetables. Maize is usually sold as grain in the local markets, traded with buyers from other parts of the country, and from Uganda (USAID, 2015).

In Kirinyaga County, our study was situated in the western and central regions of Kirinyaga, roughly 109 km from Nairobi. In 2019, 19% of the population of Kirinyaga lived below the poverty line (Kenya National Bureau of Statistics, 2022). Seventy-seven per cent of the population in the county lives in rural areas (The Kenya Ministry of Agriculture Livestock and Fisheries, 2018b) and in 2016, six per cent of households were food insecure with 18% of stunting children under five (World Food Program, 2016). Dairy and crop production, poultry keeping, and fish farming are important economic activities in the county. Kirinyaga’s proximity to Nairobi, along with the access to irrigation, makes it a nationally important maize producing area. The main cash crops include rice and horticulture in the lower areas, and coffee, bananas, and avocados in the higher areas (County Government of Kirinyaga, 2018). Maize is usually planted twice per year under irrigation and rainfed conditions. Maize is commercialised as grain in the local markets, as green for the market in the capital, and as forage for silage.

### Sampling strategy

The participants in the two study areas were selected through a three-stage sampling technique, ensuring a gender balance of 50% women and 50% men. After the selection of Kakamega and Kirinyaga, for the second selection stage, sub-counties and villages were purposively selected to ensure that participants could have access to the maize seed products in the study through the local agro-dealer or local market during the long rainy season of 2023. Finally, on the day of data collection and in each village, two teams of two researchers took paths in different directions. They approached homesteads along their path and asked the people present if they were available for an interview. They interviewed one person per homestead. When reaching the maximum number of male or female persons determined for the sampling area, they would either suggest interviewing another person or move on to the next homestead. In total, we interviewed 82 participants: 43 in Kakamega and 39 in Kirinyaga. All participants met the following conditions: (i) had grown maize in the last long-rainy season 2023 without irrigation, (ii) were actively involved in maize production and decision making, and (iii) had not received maize seed samples during the earlier (2023) long-rainy season.

### Data collection

The data collection team received a four-day training on the interview procedure and pre-tested the research tool in April 2023. Data collection took place from May to July 2023 by two teams of two researchers and two assistants. They recorded data manually. The interviews were in English, Swahili, Luhya, and Kikuyu, lasted approximately an hour and a half, and consisted of three sections. The first section registered individual and household demographics. The second section gathered information at the maize-farm level on varietal use, seed sourcing practices, and end-uses of maize (data not presented in this study) using a participatory mapping exercise. The third section applied the means-end chains (MEC) method explained below.

### Means-end chains to understand seed choices

The means-end chains methodology originates from marketing research, where it has been used to examine how consumers assess products and services and why they value them (Grunert et al., 1995; Gutman, 1982; Reynolds & Olson, 2001). Grounded in Kelly’s (1955) psychology of personal constructs, MEC proposes that human decision-making is hierarchically structured. It connects product attributes to the goals and needs that individuals seek to satisfy and, ultimately, to their personal values. By investigating *attribute–consequence–value ‘chains’*, the approach aims to reveal the motivations underpinning preferences (De Ferran & Grunert, 2007; Gutman, 1982; Reynolds & Olson, 2001).

In doing so, MEC does not directly identify the product that consumers like most*–* in our case, a maize seed product, but instead (1) examines how individuals (farmers) identify attributes (traits) to differentiate between maize seed products and (2) how these attributes serve as ‘*means*’ to the fulfilment of specific ‘*ends*’. The strength of MEC lies in the combination of qualitative depth (capturing rich individual reasoning without being directive) with a quantitative structure (aggregating across respondents), enabling to uncover the motivational structures that drive decision-making.

We applied MEC in six steps: (1) an elicitation technique to identify the attributes which individuals used to differentiate between maize seed products, (2) a rating of the elicited attributes to assess their relative importance, (3) laddering interviews to probe why attributes matter, (4) coding responses into attributes, consequences and values using content analysis to build chains or ‘ladders’ (Reynolds & Gutman, 1988), (5) compiling individual chains into an implication matrix and (6) visualisation of collective chains into Hierarchical Value Maps (Grunert et al., 1995; Reynolds & Gutman, 1988). The empirical application of each of these steps is explained in more detail below.

### Attribute elicitation and rating

**Fig. 2:**
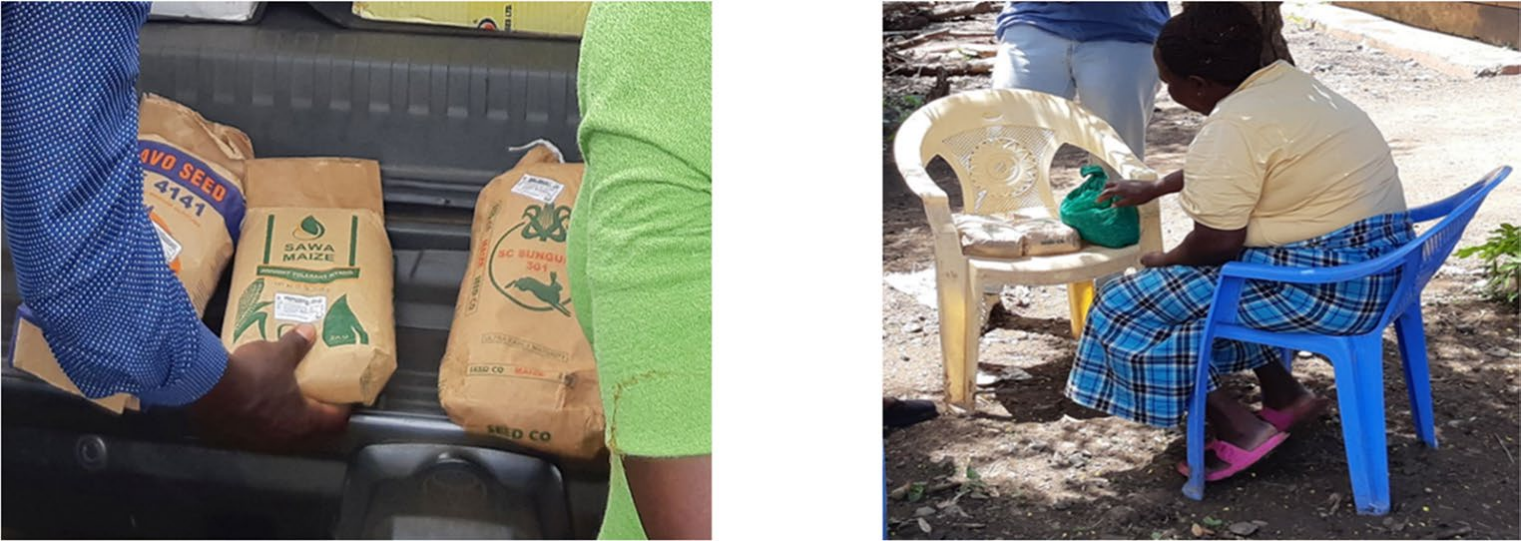
Triadic sorting exercise with farmers using sampled maize seed products in original packaging

We used a triadic sorting technique based on Kelly’s repertory grid (Kelly, 1955) as the elicitation method. We used a portfolio of seven maize seed products: six seed products in their original 2 kg packaging containing seed of hybrid maize varieties and one seed product in a common non-woven polypropylene plastic bag containing seed of the most popular open-pollinated variety (OPV) in the area, usually known as *local variety* (Fig. 2). The set of maize seed products used varied with respect to the origin of the seed company (two national, one regional and two multinational), the variety, the year of release in Kenya (from 1995 to 2016; Table 2) and the type of packaging (paper bags, plastic bags, images, and information on the package). Seeds of the hybrid maize varieties were commercially available at the agro-dealer shops at the start of the 2023 long-rainy season at a price between 500 and 750 KES, while seeds of the local OPV maize varieties could be bought at the local market for around 200 KES.

**Table 2:** Characteristics of the maize varieties used in the two study areas

Kakamega		Kirinyaga		Maize variety description
Commercial name	Year of release	Commercial name	Year of release	
Opapari	-	Makueni	-	Local open-pollinated variety (OPV)*
H513	1995	H513	1995	Hybrid maize variety, national company
DK8031	2003	DK8031	2003	Hybrid maize variety, multinational company
DK777	2016	DK9089	2012	Hybrid maize variety, multinational company
WH505	2003	Duma43	2004	Hybrid maize variety, national or regional company
WH101	2010	Sungura301	2015	Hybrid maize variety, national or regional company
Pioneer30G19	2006	Pioneer3253	1996	Hybrid maize variety, multinational company and flagship product

Source: (KEPHIS, 2023)

*The local OPV’s were the most commonly available varieties in both study areas

Each participant was presented with five different triplets of maize seed products using a randomised combination of the seven maize seed products. Each time the participant was presented a triplet of maize seed products, she or he was read the scenario below:

‘‘*Imagine that the village elder introduces you to three families. This package will be Family 1, this package will be Family 2, and this last package will be Family 3. The village elder then asks your advice on choosing two families to live together in a boma. The third family will live apart in another boma. The families that you will choose to live together must be somewhat similar. Which two families would you choose to live together due to their similarities, and which family would you send to live in a separate boma because of its differences from the other two?*’’

Participants were then asked to group two maize seed products that were more similar to each other than to the third one. Once participants had grouped the maize seed products, they were asked to explain why the two maize seed products grouped were more similar as compared to the third one. An example of the responses would be: “*these two varieties have shorter maturity time versus the third one*”. This exercise resulted in a word-pairs: at one end would be ‘*shorter maturity time’* and at the other end ‘*longer maturity time’*. To ensure that participants did not overlook any important attributes, we asked whether there were additional attributes they considered important when choosing maize seed to plant for the long-rainy season. These attributes were also considered for the laddering. Thereafter, and for each word-pair, participants choose which of the two opposite ends they preferred when selecting a variety to be planted during the long rainy season. This resulted in a list of more-valued *constructs* and less-valued *contrasts*. To understand the importance of the *constructs* when choosing a seed product for planting, participants rated their importance on a scale of one (‘*not at all important)* to five (‘*very important’)*.

### Laddering interviews

We used a soft laddering technique to understand why respondents valued an attribute. In a soft laddering approach, the respondent’s natural flow of speech is restricted as little as possible. This allows participants to freely verbalise the links between different attributes and the personal and meaningful consequences (Costa et al., 2004; Grunert et al., 1995; Reynolds & Gutman, 1988; Reynolds & Olson, 2001). In doing so, starting from each ‘construct’, participants were continuously asked “*Why is it important to you that a maize variety is/has this* ‘*construct*’?”.

### Coding and analysis

After data collection, we performed a content analysis of all the elements from the individual chains (Reynolds & Gutman, 1988). The coded word-pairs and laddering responses were classified into attributes, consequences, and values. Coding sought to group identical responses without losing relevant meaning (ibid.). Afterwards, we compared, discussed, and adapted the coding where needed.

Coded data were analysed by using the Microsoft Excel based ‘MECAnalysisTool’ (Foolen-Torgerson & Kilwinger, 2020). This tool applies a number-of-respondents-based algorithm to ensure the counting of the number of respondents who make a particular link (Kilwinger & van Dam, 2021). By aggregating individual chains, the tool produces collective links which form the chains. This quantitative structure forms the empirical foundation for constructing *Hierarchical Value Maps* (HVMs), which present the chains visually. The result is a structured yet exploratory method that blends qualitative depth with quantifiable patterns.

Microsoft Excel plugin NodeXL (Smith et al., 2010) was used to visualise the HVMs. We applied different cut-off levels. A cut-off level indicates the minimum number of participants who referred to the same link between *attributes-consequences-values.* In our analysis, the cut-off levels varied: they depended on the sample size of each group and determined on the principle of presenting as many links as possible while ensuring interpretability (Grunert et al., 1995). To further visualise the importance of different *attribute–consequence–value* chains, we adjusted the thickness of lines according to the number of respondents who referred to the specific link. Furthermore, we used colours to make it easier to distinguish the different chains in the map.

## Results

### Participants characteristics

Table 3 summarises the characteristics of the 82 participants whom we interviewed. In both counties, participants allocated the largest part of their cultivated land to maize. Participants in Kakamega planted smaller areas of maize in the short rainy season than in the long rainy season. Most respondents (60%) reported growing maize for both household consumption and sales.

**Table 3:** Demographic characteristics of the interviewed participants and their maize production and consumption during the LRS 2023

	Kakamega		Kirinyaga		Overall
	Women	Men	Women	Men	
	(*n* = 24)	(*n* = 19)	(*n* = 17)	(*n* = 22)	(*n* = 82)
Age (years)	43	47	42	46	45
Agricultural sales as the first source of income (%)	46	68	41	45	50
Average land cultivated (ha)	0.64	0.73	0.56	0.55	0.64
Average maize cultivated (ha)	0.49	0.45	0.40	0.39	0.45
Number of maize plots	2	2	1	2	2
Years managing own maize plots (years)	15	18	18	17	17
**Household consumption**					
Grain consumption (%)	92	100	94	82	91
Green consumption (%)	79	89	82	73	80
Fodder/silage use (%)	42	58	71	73	60
**Sales**					
Grain sales (%)	50	42	53	50	49
Green sales (%)	-	-	-	18	5
Fodder/silage sales (%)	-	-	29	9	9
**Other use**					
School fees and/or feeding program (%)	33	58	6	5	26
Donations to church or mosque (%)	63	74	59	45	60
Other use (%)	25	05	12	41	22
	**88**	**68**	**59**	**77**	**74**

### Attributes used to differentiate among maize seed products and their importance

Findings showed that respondents collectively used 74 word-pairs to differentiate among the seven maize seed products used in each study area. Respondents referred to seed quality differences only 10 times and to brand or packaging 11 times (Table 4). In total, 39 different word-pairs were elicited by at least four participants. The number of elicited world-pairs per respondent ranged from two to 19, with an average of 9.

**Table 4:** List of 39 word-pairs generated during the elicitation process with the number of times each pole was preferred by participants and their average importance given by participants (*n* = 82). Only those word-pairs used by four or more participants are shown in the table

Category	Construct	Times preferred	Average importance	Contrast	Times preferred	Average importance
Knowledge	Less known	2	3	More known	28	4
Packaging	Non-packaged seed	0	−	Packaged seed	4	4
	Different brand	0	−	Same brand	7	3
Certification	Non-certified seed	0	−	Certified seed	4	4
Maturity	Earlier maturity	57	4	Later maturity	12	4
Yield	Lower yield	0	−	Higher yield	66	5
Seed characteristics	Hybrid	6	4	Non-hybrid	1	3
	Lower germination rate	0	−	Higher germination rate	6	5
	Smaller seeds	2	3	Bigger seeds	5	4
	Non-pre-treated seed	0	−	Pre-treated seed	5	5
	Lower seed price	8	4	Higher seed price	4	3
Plant characteristics	Shorter plant	22	3	Taller plant	19	3
	Less robust plant	1	2	More robust plant	14	4
	Thinner stem	0	−	Thicker stem	11	4
Cob characteristics	Smaller cob	2	5	Bigger cob	31	4
	Incomplete cob filling	0	−	Complete cob filling	4	3
	Single cobbing	0	−	Double cobbing	10	4
	Less cob rows	0	−	More cob rows	9	4
	Cob does not fold	1	3	Cob folds	9	4
	Open husk cover	0	−	Closed husk cover	19	4
Grain characteristics	Smaller grain	1	3	Bigger grain	30	4
	Lower grain humidity	4	3	Higher grain humidity	0	−
	Lighter grain	2	3	Heavier grain	24	4
Tolerance and resistance	Less drought tolerance	2	3	Higher drought tolerance	32	4
	Less wind resistance	1	1	More wind resistance	4	4
	Less pre-harvest pest resistance	0	−	More pre-harvest pest resistance	22	4
	Less post-harvest pest resistance	0	−	More post-harvest pest resistance	27	4
	Less resistance to diseases	0	−	More resistance to diseases	4	4
Input requirement	Less rain requirement	28	4	More rain requirement	22	4
	Less fertiliser requirement	34	4	More fertiliser requirement	4	3
Suitability to season	Suitability to short rainy season	1	2	Suitability to long rainy season	4	4
	Suitability to one rainy season	0	−	Suitability to two rainy seasons	6	3
Market demand	Less green market demand	4	2	More green market demand	13	3
	Less fodder market demand	1	2	More fodder market demand	5	4
	Less grain market demand	0	−	More grain market demand	6	4
Culinary characteristics	Easier to shell	7	3	Difficult to shell	0	−
	Shorter shelf-life	0	−	Longer shelf-life	4	4
	Less white flour/ugali	0	−	Whiter flour/ugali	5	3
	Lighter flour/ugali	2	3	Heavier flour/ugali	25	4
	Less tasty	5	2	Tastier	34	3

* For ease of interpretation we considered a construct the lower word-pair and a contrast the higher word-pair.* Average importance: 1 = Not at all important, 2 = Slightly important, 3 = Important, 4 = Fairly important, 5 = very important

Participants predominantly referred to attributes that were variety characteristics. In general, participants seemed confident about the attributes of the maize varieties and differences between them. Some participants hesitated, however, when presented with packages of two different hybrid maize varieties from the same brand/company within a triplet. This was in particular the case with the Dekalb hybrid varieties, which only differed in the numerical code of their name (see Table 2). This is in contrast with the maize seed products of other companies. Western Seed uses different names for its hybrid maize varieties, while Seed Co uses different names and unique packaging. Twelve participants mentioned the price of seed as a consideration: eight farmers preferred the costlier seed product, four farmers the cheaper seed (Table 4). Seven participants grouped seed packages based on brand, and four distinguished between packaged and seeds sold in plastic bags. Four participants differentiated between certified and non-certified seeds, preferring the former, and six participants who referred to the germination capacity of the seeds.

The attribute elicitation exercise (Table 4) proved that *higher yield* (*n* = 66), was the attribute most often referred to, followed by *earlier maturity* (*n* = 57), *less fertiliser requirement* (*n* = 34), *tastier* (*n* = 34) and *higher drought tolerance* (*n* = 34). The attributes that participants most often referred to were not always granted the highest level of importance. For example, almost half of the participants differentiated the varieties in the study on the basis of *taste* with the majority preferring *tastier* varieties (*n* = 34) over *less tasty* varieties (*n* = 5). However, the importance of *tastier* varieties scored only 3 on a scale of 5. Likewise, participants in our study preferred both *shorter plants* (*n* = 22) and *taller plants* (*n* = 19) but considered the tallness of the plant 3 on the scale of 5. In contrast, only a few participants referred to the attributes of *higher germination rate* (*n* = 6), *pre-treated seed* (*n* = 6), *and smaller cob* (*n* = 2); however, those who did, rated them as ‘very important’ (5 on the scale of 5). These results suggest that even if certain attributes are frequently used to differentiate among varieties and preferences are obvious (i.e. taste), it does not necessarily mean that these attributes are highly important when choosing what seed to use for planting.

### Relating attributes to consequences and values

**Fig. 3:**
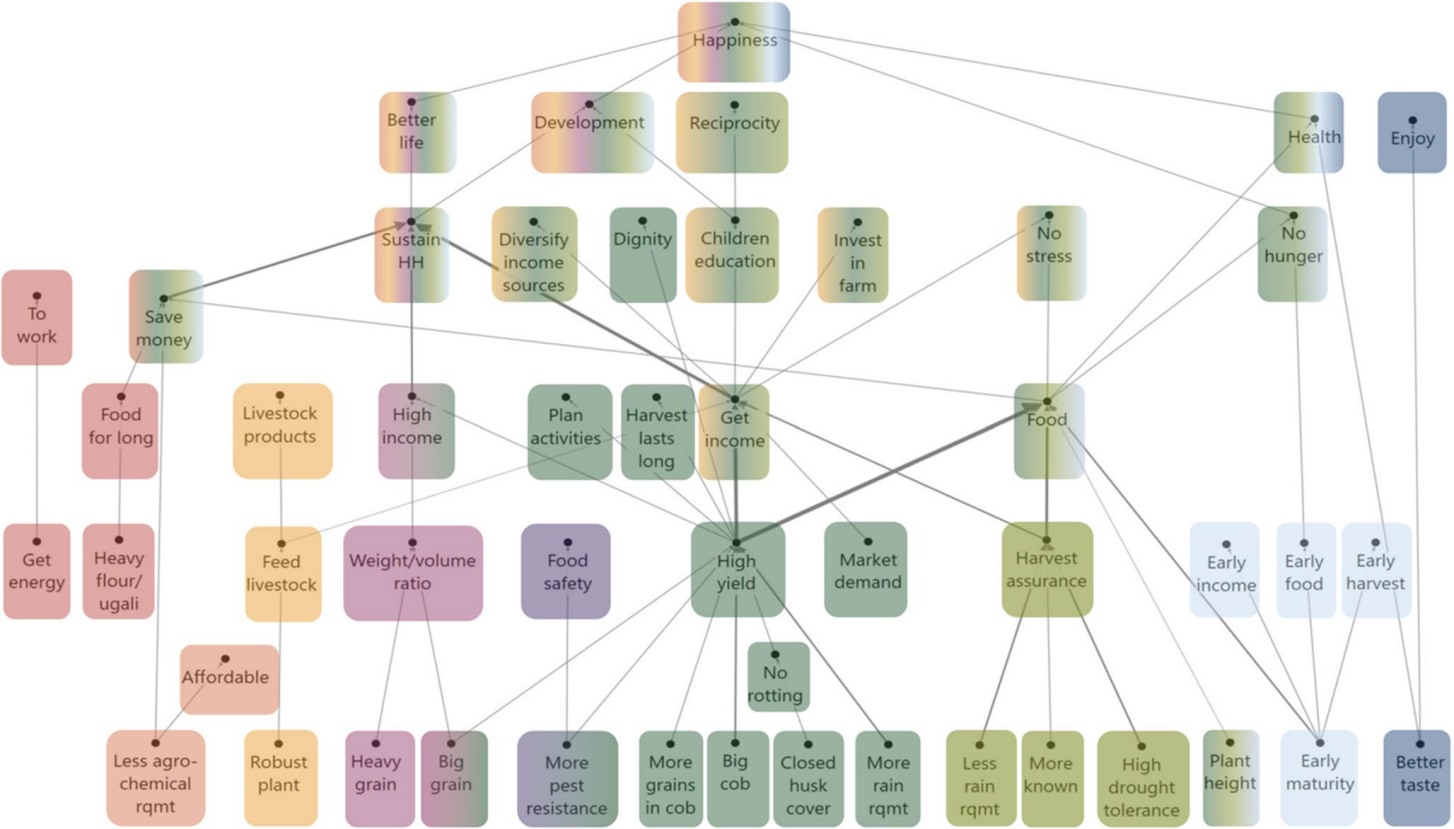
HVM of the whole sample (*n* = 82), with cut-off level *n* = 10. Different colours show the chains related to different consequences. Direct links are shown by solid lines, and indirect links by dotted lines

We used a cut-off level of 10 to create the overall hierarchical value map (HVM). This means that only linkages made by 10 or more respondents are presented in the HVM, corresponding to ≥ 12% of the total number of participants. The overall HVM (Fig. 3) shows 15 out of the 74 attributes that participants used to differentiate among maize varieties.

A first chain, presented in red in Fig. 3, relates to the **quality of maize produced for home consumption**. Participants in our study preferred maize that provides them with sufficient *energy to work* and *heavy flour* and *ugali*. They explained that when maize flour is heavy, it is consumed in smaller quantities per meal and the *food lasts for longer* in the household, which in turn *saves money* that can be used otherwise to *sustain the household*.

A second chain, presented in orange, is related to *the agrochemical requirements* of the maize and **affordability**. Participants often mentioned they could not afford the inputs that hybrid maize varieties need and therefore prefer to use varieties with lower input requirements. A low agrochemical requirement also allows participants to *save money* that can then be used for other household expenses.

A third chain, presented in yellow, relates to **livestock**. Participants preferred *robust maize plants* to feed their livestock with the harvest residues and obtain livestock products. This cluster portrays the multidimensional role of maize in rural livelihoods and the importance of fodder and livestock.

A fourth chain, presented in pink, relates to the **weight-volume ratio** that results from the attributes of *bigger size* and *heavier weight* of the maize grains. Their importance relates to the two ways in which maize grain is commercialised in the study areas: per ‘*gorogoro’* or per kg. People commercialise small quantities of grain locally per *gorogoro*, a volume of a standard tin, which is traditionally used to sell 2 kg of cooking fat. Sellers prefer *bigger grain* because it fills the tin with fewer grains. When maize is sold by weight, *heavier grain* is preferred because fewer grains are needed for a kilo. These two grain attributes were considered important to get a desired *weight-volume ratio* that leads to a *higher income* to *sustain their household* and achieve a *better life*.

A fifth chain, presented in purple, relates to **food safety**. Participants related maize varieties that are *resistant to pests and diseases* to *food safety*. In Kenya, maize is heavily affected by pests and diseases in both the pre-harvest and post-harvest stages. The use of pesticides and fungicides during production and storage phases is a food safety concern.

A sixth chain, presented in dark green, relates to **high yield**. The HVM shows that this is the most important chain. Participants preferred six attributes because they lead to a *higher yield*: *bigger grain size*,* higher pest resistance*,* more grains in cob*,* bigger cob*,* closed husk cover* and *higher rain requirement*. From a *higher yield*, participants could get *food* and *income*. Next to more *income* and more *food*, participants said they pursued high yields for a *long-lasting harvest*, to carry out their *activities as planned*, and to maintain their *dignity*. They also mentioned that if the harvest lasts, they can sell their produce later and sell for a higher price in the *market*. Participants considered that they could maintain their dignity by not having to lend money or plunder the maize fields of others.

A seventh chain, presented in light green, relates to **harvest assurance**. This chain originates from three attributes: *less rain requirement*, *more known* (i.e. they were more familiar with the varietal characteristics and management practices) and *higher drought tolerance*. Harvest assurance is a main link to *food* and *income*, like high yield. With food in the household, there will *not be hunger*, participants will *not be stressed* and will be *happy*. Participants mentioned different goals for wanting an income from maize: they could *diversify their income sources*, invest in the farm, sustain the household, and *pay children’s education*. *Sustaining the household* was central for participants to have a *better life* and to achieve *development* and *happiness*. *Children’s education* was considered important for their children to be *developed* and to be *reciprocated* by them in the future.

An eighth chain, presented in light blue, relates to **earliness.** Although participants were interviewed about their preferences for the long-rainy season maize, many participants said they prefer *early maturing* varieties over *long maturing* varieties. By planting early maturing varieties, they secure *early harvest*, *early income*, and *early food*. By having *food early*, the household will *avoid hunger*, which contributes to their food security. From anecdotal data we infer that households in our study are increasingly challenged by the effects of climate change on maize production, such as a delayed start, the intensity and frequency of the rains. Moreover, many said they experience more or less acute food shortage at the end of the dry season and the first part of the growing season, typically referred to as the ‘hunger gap’. As a result, food expenses in that period increase while there is also the need to pay school fees and/or contribute to school-feeding programs at the start of the school year. Consequently, early maturing varieties can ease the pressure on the household’s economy.

A final chain, presented in dark blue, relates to *good taste* and **enjoying** food. Participants affirmed that they could distinguish between varieties based on the taste of roasted maize, porridge, ugali and *githeri*.

### Differences between Kakamega and Kirinyaga

**Fig. 4:**
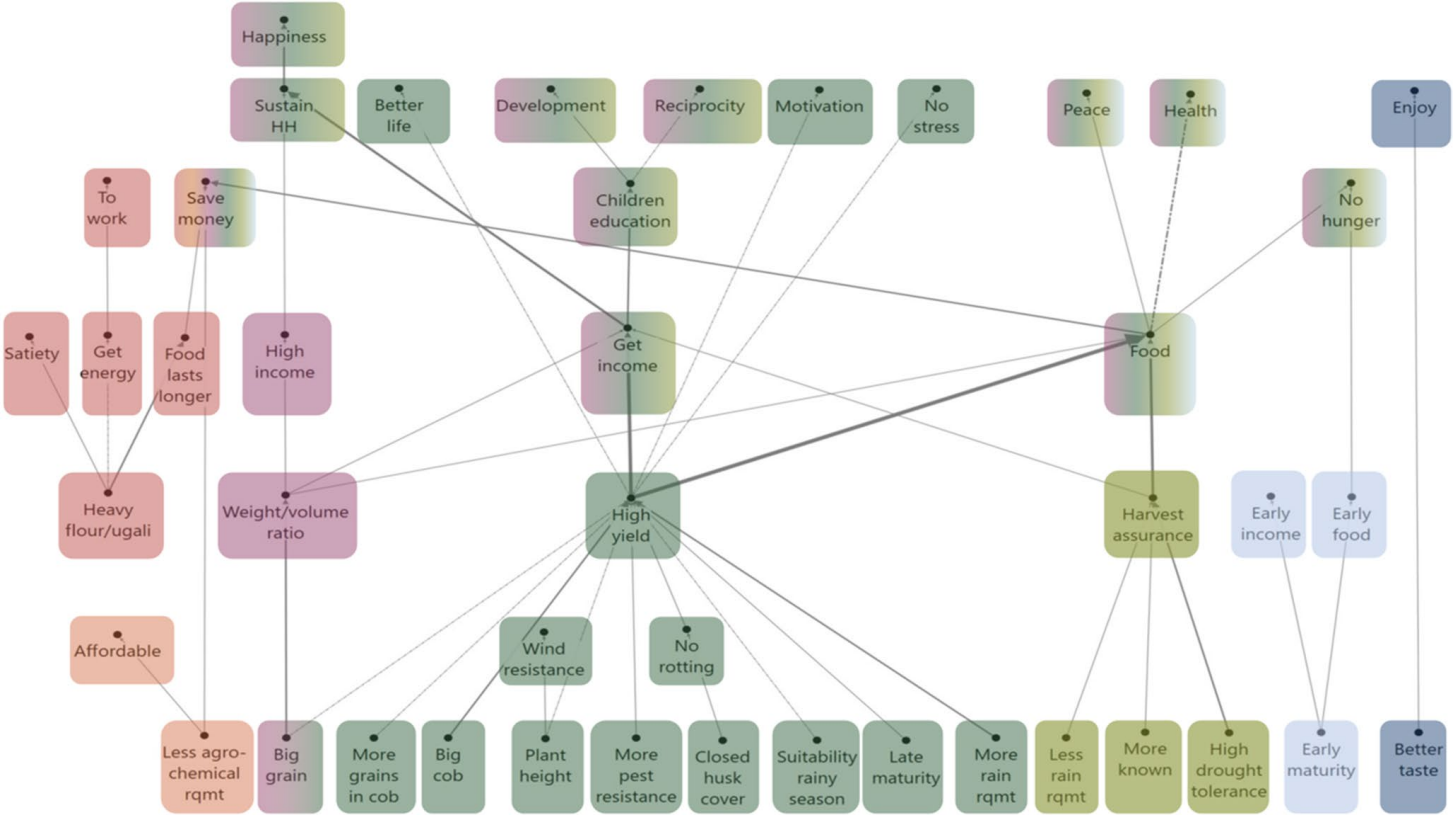
HVM of Kakamega (*n* = 43), cut-off level *n* = 6. Different colours show different chains related to consequences. Direct links are shown by solid lines, and indirect links by dotted lines

**Fig. 5:**
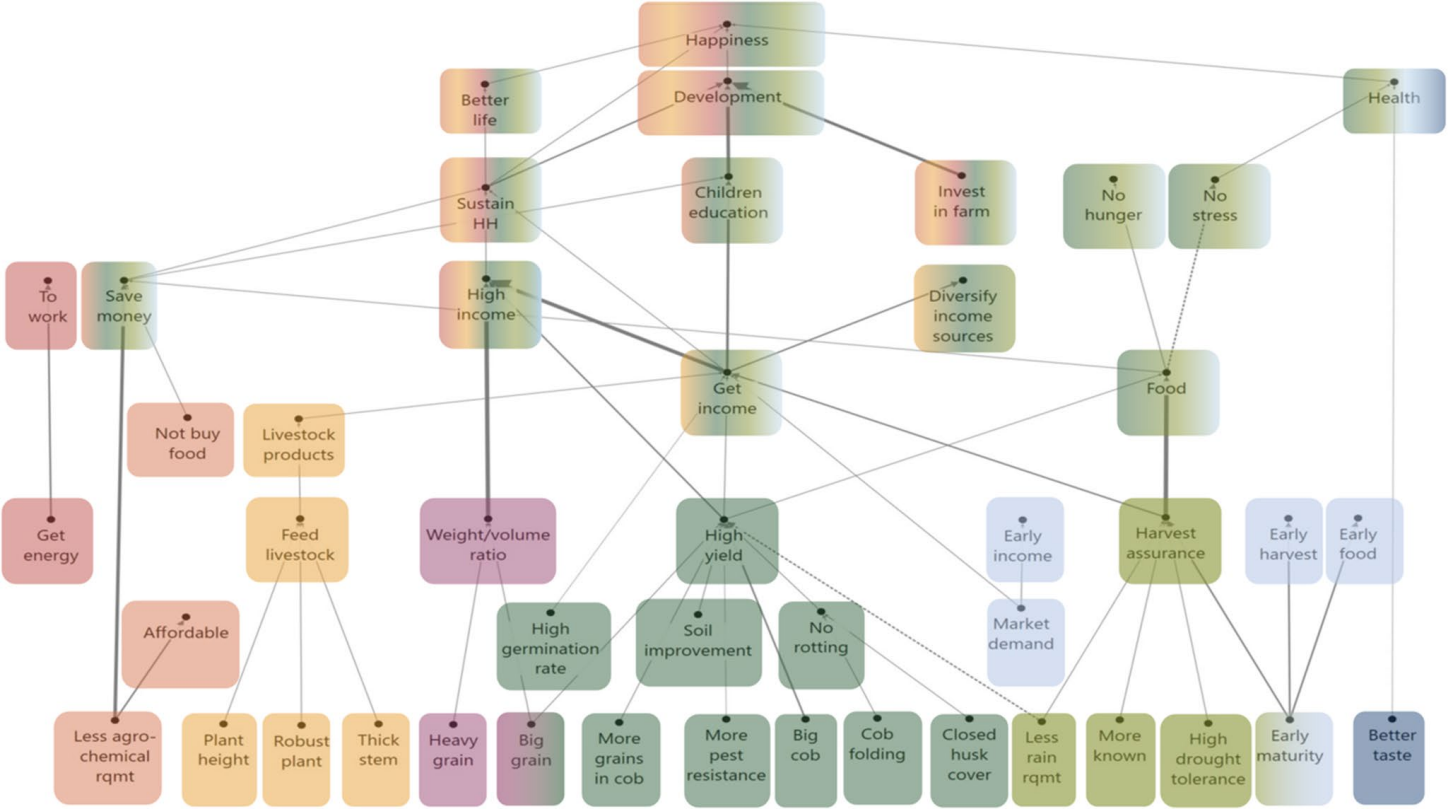
HVM of Kirinyaga (*n* = 39), cut-off level *n* = 6. Different colours show different chains related to consequences. Direct links are shown by solid lines, and indirect links by dotted lines

The HVMs of Kakamega (Fig. 4) and Kirinyaga (Fig. 5) show that participants in both areas used similar numbers of attributes to differentiate between the maize seed products (15 and 16, respectively), although the attributes themselves and chains leading from them differ. The HVM of Kakamega shows more differentiation in the chains related to the **quality of maize produce for home consumption** than the HVM of Kirinyaga. Participants in Kakamega value *heavier maize products* in the form of flour and ugali to get a higher *sense of satiety*, to *get energy to work*, and to have *food for longer* to *save money* in the household. Participants in Kirinyaga only related *get energy* from maize to be able *to work*. Additionally, the presence of the **livestock** chain in the Kirinyaga’s map forms an important difference between the study areas. This chain appeared from a group of three attributes: *plant height*, *thick stem*, and *robust vegetation* from which participants expected to *feed their livestock*, g*et livestock products*, and *get an income*. The importance of this chain can be explained by the more prominent presence of mixed crop–livestock systems and/or the lack of any fodder alternatives for the livestock in Kirinyaga.

Our analysis shows differences in the consideration of the **weight-volume ratio** of the maize grains. While participants in Kakamega preferred b*igger grain*, in Kirinyaga, *bigger grain* was equally important as *heavier grain.* This reflects a difference in the ways maize grains are commercialised. In Kakamega, maize grain is mostly sold to local mills, while in Kirinyaga, maize is sold in two ways: i.e. dried grain sold to milling companies and fresh maize for the urban green market mainly in Nairobi.

**High yield** is an important chain in both HVMs, but different attributes lead to it. In both study areas, farmers consider *bigger grain*, *more grains in the cob*, *closed husk cover* and *more pest resistance* crucial for getting a *higher yield*. However, in Kakamega, participants also preferred *bigger cob*, *plant height*, *suitability to the rainy season*, *late maturity*, and *more rain requirement*. In contrast, participants in Kirinyaga considered a *high germination rate* and *improving the soil* (from foliage that can be used to cover the soil, or to feed livestock to get manure) important to get a *higher yield.* Moreover, Kirinyaga participants also preferred *cob folding* because it helps to keep the green maize fresh when it is transported to the urban market.

Similarly, while **earliness** is present in both HVMs, there is also a difference. In both study areas, *early maturity* is an important attribute for an *early harvest* and *early food*. However, participants in Kirinyaga also favour early maturity to meet *market demands*, which is important to get an *early income* in the household.

Lastly, while in both study areas **tasty** maize is valued, participants in Kakamega valued *tasty* maize to *enjoy*, while in Kirinyaga they associated taste with *health*. Participants in Kirinyaga gave two reasons for the link between taste and health. First, according to participants, the industrial milling process makes packaged flour less nutritious and less tasty. Second, it is a common practice to apply pesticides to the stored dried grain to preserve it, but this notably affects the taste. Thereby, in both cases, a *tasty* maize is a proxy for *healthy* maize.

### Gendered differences

**Fig. 6:**
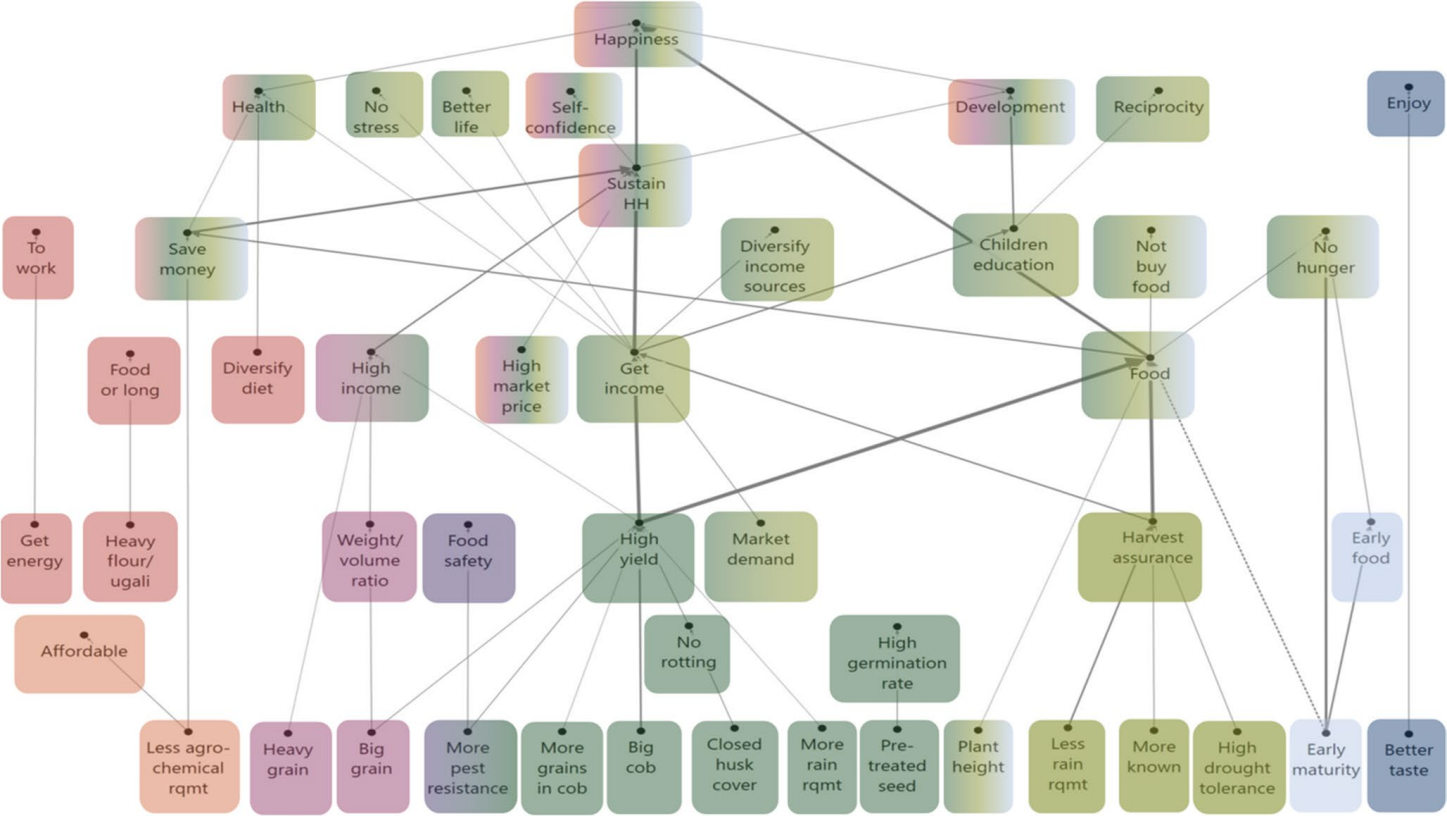
HVM of women (*n* = 41), cut-off level *n* = 6. Different colours show different chains related to consequences. Direct links are shown by solid lines, and indirect links by lines

**Fig. 7:**
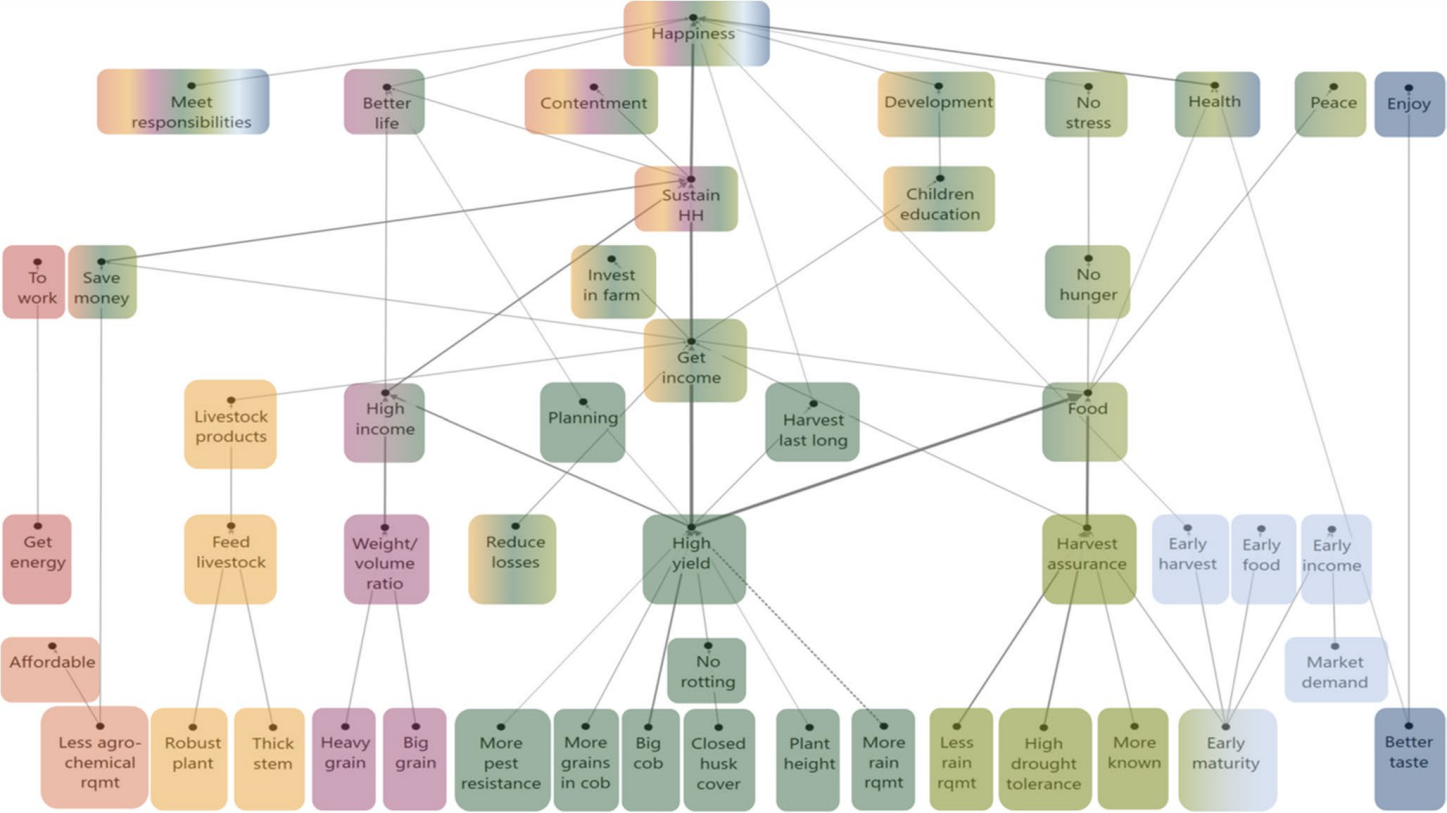
HVM of men (*n* = 41), cut-off level *n* = 6. Different colours show different chains related to consequences. Direct links are shown by solid lines, and indirect links by lines

The HVM of the women (Fig. 6) shows a similar number of attributes (15) as the HVM of the men (16) (Fig. 7). There are, however, differences in the chains at the attribute, consequence and value levels.

In the chain related to the **quality of maize produce for home consumption**, both men and women related *get energy* from maize to be able *to work*, yet women’s HVM shows greater detail in this chain. Women expressed a preference for maize that produces *heavier flour and ugali* to have *food for longer* in the household. They also emphasised that having a *diverse diet* is important for them to be *healthy*. This suggests that the women in our study found various aspects of food security very important, in particular the availability of sufficient food quantities and dietary diversity for the household.

Another important difference is that only the men’s HVM shows a **livestock** chain. This chain starts from two attributes: *thick stem* and *robust vegetation*. From these attributes, the men in our study expected to be able to *feed livestock*, to get *livestock products* and to get *an income*.

Women in our study considered it important to select a variety with *pest resistance* in order to increase **food safety** and get **higher yield**, while men related *pest resistance* only with **higher yield**. Several women mentioned that the application of pesticides to protect the stored maize had a negative impact on health. Possibly, women are more aware of pest and diseases affecting the maize during storage and have a more nuanced understanding of for example contamination with mycotoxins and toxigenic fungi in storage.

Despite HVMs of women and men displaying both **high yield** and **harvest assurance** chains, they stem from different maize attributes. The first difference at attribute level is that women preferred *pre-treated seeds* for getting a *higher germination rate*. Because women often do the planting and weeding, they may observe that sometimes non-treated seed lots do not germinate well. Furthermore, both men and women valued *high yield* for *high income*, *income* and *food*, and *harvest assurance* for *income* and *food*, but women valued *income* in order to *diversify income sources*, whereas men considered *income* important to *invest in the farm*. These findings might relate to the gender roles in the rural household: women are more often a small family business, such as a corner shop or selling food, while men oversee the farm and livestock production.

Both women and men in our study preferred **early maturing** varieties over late maturing ones. However, women valued **early maturing** varieties to get *early food* and to *avoid hunger.* Men, in contrast, expected an *early harvest*, *early income*, *early food*, and *harvest assurance* from this attribute. Also, women and men preferred **tastier** varieties over less tasty ones. Both desired tastier maize varieties to **enjoy**, but men related tasty maize also to *health.*

Finally, at the value-end level of the chains, women and men showed considerable overlap, both emphasizing values such as *enjoyment*,* sustain the household*,* better life*,* children education*,* to work*,* development*,* no stress*,* health*,* and happiness.* These are values that both groups derive from growing maize with desired attributes. However, there are specific values for each gender. Women said they draw *self-confidence* from maize production and hope for future *reciprocity* from their kids. Men said they feel *contentment* and *peace and* considered growing maize a way to *meet their responsibilities* as household heads.

## Discussion

### Differentiating between maize seed products

A relevant finding from the triadic sorting exercise was that participants used a wide range of attributes to differentiate among the maize seed products used in our study. It is important to note that we did not use the most recently released hybrid maize varieties (that is, we used varieties that were released four years ago or more): we used a portfolio of common varieties that were readily available in the agro-dealer shops in the study areas. Nevertheless, our findings challenge the common proposition that smallholders lack knowledge about the available maize seed products. On the contrary, farmers knew and evaluated the maize seed products on the basis of many attributes, which suggests they are very familiar with the variety portfolio available to them. This finding calls for a deeper understanding of ways in which smallholders familiarise themselves with new hybrid maize varieties (e.g., own experimentation and experience, neighbour’s plot, demonstration plots) and how this might influence their seed choices. The findings also point to the need to better understand the challenges of the other actors in the value chain, such as seed companies, agro-dealers and governments, in relation to seed availability and knowing about farmer preferences.

Furthermore, results show that participants hardly elicited attributes related to the packaging and, unlike earlier findings in this area (Rutsaert et al., 2024). Our results suggest limited brand/company loyalty. The price of the maize seed did not appear as an important consideration for farmers either. We noticed farmers got confused when presented with a triplet that included two hybrid maize varieties from Bayer Dekalb while this was not the case when they were presented with two hybrid maize varieties from Western Seed Company or Seed Co. This suggest that the use of different names, colours and logos on the seed packages help smallholders to differentiate among the available hybrid maize varieties and thereby make better and informed seed choices. Altogether, these findings have implications for product marketing and call for further studies to better understand how different seed companies and brands are perceived by smallholders and under which conditions farmers show company/brand loyalty.

### The most preferred attribute might not always be the most important

The attribute rating exercise gave us two important insights. First, it showed that in certain cases the most frequently mentioned attributes to differentiate between maize seed products were not considered the most important ones when choosing a seed to purchase. This was for example the case with taste. Second, some participants had opposing preferences for several other attributes, such as rain requirement, maturity time. Thus, while some participants preferred late maturing varieties, others preferred early maturing ones. And, some liked varieties that did well with little rain while others said they liked the ones that did well with abundant rainfall. Because we asked farmers about their preferred seed attributes in the long rainy season, we cannot rule out that these preferences may be different in the short rainy season.

### Different varieties for diverse needs

The HVMs confirmed the multi-purpose importance of maize as staple crop for food security in the household, as a cash crop to support the household economy and as fodder to feed livestock. Furthermore, while food, feed, and income are primary goals of maize production, our analysis shows other nuances, highlighting the importance of the timing, quantity, and reliability of the maize being available to the household. Our results show that smallholders fulfil household needs through maize production and that they are not achieved through maximising yield only. Although participants desired high yield, our results stress the importance of early maturing varieties for ensuring *early harvest*,* early food*,* early income* and *harvest assurance* in the household. Also, attributes like taste and considerations of health are important. The findings also show that maize smallholders in Kenya have diverse needs they expect to meet through their maize production and hence require varieties that vary in the traits.

Further, preferring a more known variety was often mentioned. This is a key attribute in relation to the challenge of increasing variety turnover. If large numbers of new hybrid maize varieties are released over a short period of time, adoption of these varieties may be hindered farmers may not have sufficient time to familiarise themselves with them (Misiko, 2013; Stone, 2007).

### Different varieties for different smallholders’ groups

Differences between the two study areas were present at the attribute, consequence, and value levels of the maize seed product chains. These differences were influenced by climate characteristics, especially by rainfall patterns, the importance of livestock and specific market demands. In general, the chains reflect that maize in Kakamega has a more central role as an economic activity and as the main food staple, whereas in Kirinyaga, participants are growing maize as one of their agricultural activities and income sources. The differences between chains at attributes, consequences, and values can provide insights for breeders to develop new hybrid maize varieties that respond to the different needs of farmers and to develop strategies to market their maize seed products.

Differences between women and men were minor at the attribute level and mostly visible at the consequence and value levels. This supports other research that also found that men and women in Kenya differ little in preferences for maize seed attributes (Marenya et al., 2021). Our results also suggest that the differences at the attribute level might be linked to the different roles that men and women have in the maize production of a household. For example, men prefer attributes such as *robust plants* and *thick stems* because they might be in charge of livestock production, compared to women preferring *pre-treated seeds* because they do the planting and weeding and are taking management decisions related to planting and labour allocation (Voss et al., 2023). Our results also back up the common findings that women focus more on food-related benefits like food safety (pest resistance), food security (heavier flour/ugali and to have food for longer), and food quality (diverse diets), while men pay closer attention to the household economy. While acknowledging that there were differences at the consequence level of the chains, our study suggests that women and men pursue similar end-goals: to sustain and develop their households.

## Conclusions

This study highlights the large variety of attributes that smallholders use to differentiate between and choose among maize seed products available to them. The use of a broad range of attributes to evaluate and differentiate maize seed products shows that smallholders have knowledge about the maize varieties used in the MEC exercise and commonly available in their home area. Smallholders’ preferences for specific attributes also proved to be diverse and context dependent. Key production goals, i.e., higher yield, harvest assurance, and earliness, were consistent across groups and equally valued. These goals served various aspects of food security and well-being. Packaging and price of the seeds hardly played a role, and gender was of minor difference in the attribute differentiation. These findings suggest that smallholders are well aware of the attributes they need, and in which seed products to find them. Furthermore, our results underscore the importance for farmers of knowing a variety. This, in turn, suggests that it is important that farmers get the opportunity to gain experience about the performance of a variety in their own fields, which requires one or more seasons of observation. If farmers need time to evaluate a variety for a broad range of attributes before deciding if continuing to use it, then the pursuit of a high varietal turnover asks for farmers to have early and ample opportunities to become familiar with the new varieties. This could for example, be stimulated by handing out free samples of seed or more demo-plot trials. The effectiveness of such strategies would need to be evaluated. Lastly, the identified differences in attribute preferences and groups of attributes across smallholders offer valuable guidance for breeding programmes, enabling them to breed varieties that are not only agronomically productive but also aligned with diverse, context-specific realities of smallholder farmers. Evaluation of new to-be-released varieties against the range of attributes that farmers consider – under on-farm conditions – is another step that can contribute to better identifying suitable varieties for different areas and user groups, leading to an accelerated variety turnover. Furthermore, to better engage with smallholder farmers, seed systems actors need to step back and better understand the livelihood strategies of smallholder farmers and how their contexts shape seed choices. By acknowledging the nuances of farmer preferences and decision-making, breeding programmes and seed system actors can contribute to more inclusive, sustainable, and demand-responsive seed systems.

## Abbreviations

SSA: Sub-Saharan Africa
OPV: Open-pollinated variety
HVMs: Hierarchical Value Maps
